# Complete Genome Sequence of Halopseudomonas aestusnigri Strain GOM5, Isolated from Asphalt Marine Sediments of the Gulf of Mexico

**DOI:** 10.1128/mra.01222-21

**Published:** 2022-03-09

**Authors:** Jorge Rojas-Vargas, Ricardo González-Sánchez, Alejandro Sánchez-Flores, Alexei Fedorovish Licea-Navarro, Liliana Pardo-López

**Affiliations:** a Departamento de Microbiología Molecular, Instituto de Biotecnología, UNAM, Cuernavaca, Morelos, México; b Departamento de Innovación Biomédica, CICESE, Ensenada, Baja California, México; University of Southern California

## Abstract

We report here the complete genome sequence of a marine Halopseudomonas aestusnigri strain isolated from asphalt sediments of the Gulf of Mexico. Studying the genomes of atypical environmental bacteria increases knowledge about the biology of microorganisms metabolizing pollutants and is also a biotechnological resource to develop bioremediation methods.

## ANNOUNCEMENT

We introduce the whole genome of Halopseudomonas aestusnigri strain GOM5, isolated from asphalt-contaminated sediments from the Gulf of Mexico from 350 m deep (18.76 latitude, −94.26 longitude). The asphalt sample was obtained using a box corer-type device introduced 60 cm deep. The sample was incubated for 4 weeks in Czapek medium with 0.4% of 40 American Petroleum Institute (API) oil as a carbon source at 20°C and 180 rpm and then plated in agar with solid Czapek medium; a single colony was picked. The bacteria were grown overnight on LB broth at 30°C and 180 rpm, followed by genomic DNA extraction using the Quick-gDNA miniprep kit (Zymo Research) following the manufacturer’s specifications. For sequencing, a hybrid approach was used with Illumina MiSeq and Oxford Nanopore Technologies (ONT) MinION platforms, at the Massive Sequencing Unit of the Institute of Biotechnology UNAM. Separate DNA extractions from different inocula were used for each sequencing technology. Illumina sequencing was carried out using the Nextera library kit (Illumina, Inc.) where DNA was fragmented enzymatically, to generate an average fragment size of 500 bp. A 600-cycle sequencing kit was used to obtain 828,040 paired-end reads with a read length of 300 bp. The read quality was examined using the FastQC software v0.11.9 (1). For Nanopore sequencing, DNA was not sheared, and libraries were prepared with the SQK-LSK109 kit and multiplexed using the EXP-NBD104 barcoding kit. The libraries were loaded into the R9.4.1 flow cell. Reads were base called and demultiplexed using Guppy v4.4.1, adapters were trimmed, and barcode separation was performed by Porechop v0.2.4 (https://github.com/rrwick/Porechop). The read quality with NanoPlot v1.30.1 (2) showed 37,711 reads with a mean length of 11,694 nucleotides (nt), a maximum read length of 128,087 nt, and an *N*_50_ value of 18,892 nt.

Genome *de novo* assembly was done with Unicycler assembler v0.4.8 (3). The quality analysis of the assembly was carried out with the QUAST program v4.0 (4) and that of the completeness and contamination with the CheckM software v1.1.3 (5). The genome contains 3,996,286 bp in 1 contig and has a G+C content of 60.61%. A completeness of 100% and contamination of 0.52% were obtained, with a 125× sequencing depth with Illumina and 110× with Nanopore.

**FIG 1:**
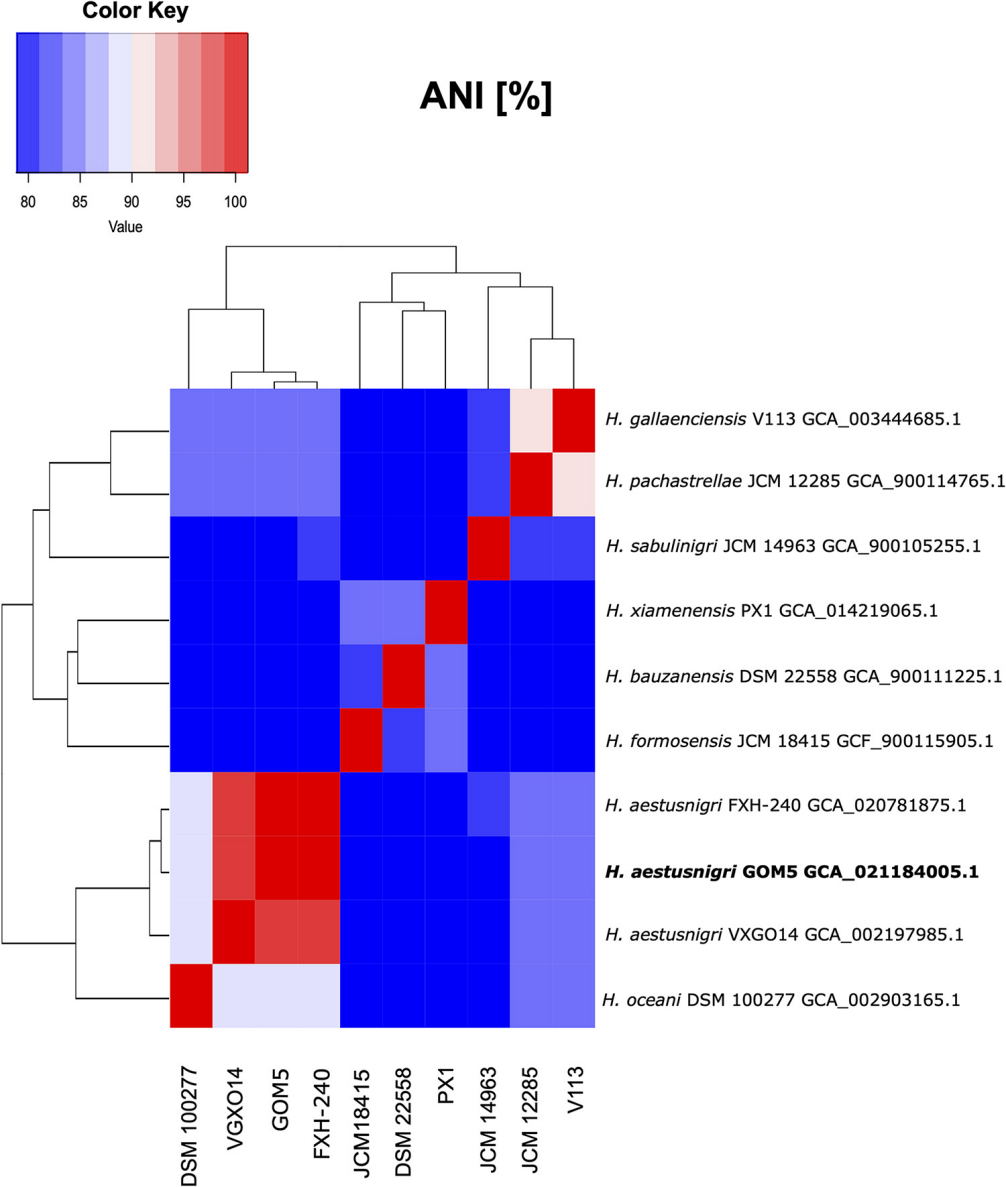
Heat map of fastANI values. Higher values correspond with greater genetic homology between strains (red). The GOM5 has 84.24% alignment with the VXGO14 genome, 87.58% with the FXH-240 genome, 65.68% with the DSM 100277 genome, and less than 50% with the other genomes.

For the first taxonomic identification, GOM5 was compared against a custom database containing type strain assemblies of the 14 *Halopseudomonas* species from the National Center for Biotechnology Information (NCBI) portal (https://www.ncbi.nlm.nih.gov/assembly, consulted on 19 November 2021), including the two available assemblies for *H. aestusnigri* (https://figshare.com/articles/dataset/Database_ANI_GOM5/19126526). The nine selected assemblies were the nearest in average nucleotide identity (ANI) to our genome. The ANI was determined with fastANI v1.32 (6) (Fig. 1). The GOM5 strain has ANI values greater than 80% with strains of the genus *Halopseudomonas* and alignment coverages superior to 80% with *H. aestusnigri*, indicating that GOM5 is strongly related to this species.

Gene prediction and functional annotation were performed with the NCBI Prokaryotic Genome Annotation Pipeline (PGAP) (7) and reported 3,735 genes, 3,585 protein-coding genes, and 75 RNAs. Default parameters were used for all software unless otherwise specified.

### Data availability.

The whole-genome sequence data and raw sequences are available at NCBI under the accession number ASM2118400v1, BioProject accession number PRJNA781385, and Sequence Read Archive (SRA) accession numbers SRX13826485 (Illumina raw sequence data) and SRX13826486 (MinION raw sequence data).
